# Statin-Induced Necrotizing Autoimmune Myositis Presenting With Progressive Limb-Girdle Weakness Following Low-Dose Atorvastatin Use

**DOI:** 10.7759/cureus.102783

**Published:** 2026-02-01

**Authors:** Layla Annous, Osama Mahmoud

**Affiliations:** 1 Internal Medicine - Pediatrics, Christiana Care Health System, Newark, USA; 2 Internal Medicine, Christiana Care Health System, Newark, USA

**Keywords:** anti-hmgcr antibody, anti-hmgcr myopathy, autoimmune necrotizing myositis, ivig, limb-girdle weakness, low-dose atorvastatin, proximal muscle weakness, statin-induced myopathy, statin-induced necrotizing autoimmune myositis

## Abstract

Statins are a mainstay in the prevention of cardiovascular disease and are associated with rare adverse effects, including statin-induced necrotizing autoimmune myositis (SINAM). This condition is characterized by progressive, symmetric, proximal muscle weakness and elevated creatine kinase (CK), persisting despite discontinuation of the statin, and is confirmed by the presence of anti-HMG-CoA reductase (HMGCR) antibodies. We present a case of a 72-year-old male who presented with three months of progressive limb-girdle weakness with chronic use of low-dose atorvastatin (10 mg). Our review of the literature suggests that this is the first documented case of SINAM in the setting of low-dose atorvastatin usage. Diagnosis was confirmed by anti-HMGCR antibodies. The patient was initially managed with corticosteroids and methotrexate but required escalation to intravenous immunoglobulin (IVIG), which led to functional improvement. This case underscores the need for high clinical suspicion for SINAM and highlights the importance of consideration for early aggressive immunosuppression.

## Introduction

Statins are lipid-lowering agents widely prescribed to reduce cardiovascular risk, with an estimated 92 million users in the United States as of 2019 [1]. While typically well tolerated, statins can cause myopathy, ranging from mild myalgias to severe necrotizing autoimmune myositis. Among these, statin-induced necrotizing autoimmune myositis (SINAM) is a rare but serious complication, affecting approximately one to three per 100,000 users [2,3]. SINAM is an inflammatory myopathy that typically presents as insidious, symmetric, limb-girdle muscle weakness associated with elevated creatine kinase (CK), similar to many other self-limiting and immune-mediated myopathies, with the defining feature being the presence of anti-HMG-CoA reductase (HMGCR) antibodies [4,5]. Unlike typical statin myalgias, SINAM results in significant muscular necrosis and functional impairment, persisting or worsening despite statin discontinuation. SINAM requires aggressive immunosuppressive therapy to achieve laboratory remission, though optimal induction and maintenance regimens are yet to be established [6]. In particular, malignancy is an independent risk factor for the development of SINAM, among other inflammatory myopathies, and appropriate cancer screenings should be completed when investigating progressive limb-girdle weakness [2]. This case highlights the rare presentation of SINAM in a patient on previously tolerated, long-term, low-dose atorvastatin (10 mg) and emphasizes the need for clinician awareness and aggressive treatment of this inflammatory process.

## Case presentation

A 72-year-old male with a history of hypertension, atrial flutter, deep vein thrombosis (DVT) and pulmonary embolism (PE), and coronary artery disease presented with a three-month history of painless, worsening lower extremity weakness and dysphagia. He had declined from independent ambulation to requiring a wheelchair. He denied trauma, recent illness, sensory loss, or myalgias. Medications included atorvastatin 10 mg daily, which he had taken without issue for over five years, metformin 500 mg twice daily, apixaban 5 mg twice daily, and hydrochlorothiazide 12.5 mg daily, with no new medications. The patient denied any smoking, alcohol, or other recreational drug use history. 

On exam, proximal lower extremity strength was 3/5, proximal upper extremity strength 4/5, with intact distal strength, preserved reflexes, and no sensory deficits or skin findings. The patient's swallow was also impaired, requiring a modified diet of soft and bite-sized/mildly thick liquids (International Dysphagia Diet 6/2). 

Labs were significant for elevated CK, alanine aminotransferase (ALT), and aspartate aminotransferase (AST), consistent with muscular necrosis. Thyroid-stimulating hormone (TSH) was noted to be elevated with normal free T4, interpreted as subclinical hypothyroidism. Suspicion was higher for SINAM than hypothyroid myopathy given only a mildly elevated TSH, and thyroid studies were noted to normalize without medical intervention after the patient's eventual discharge. Serum anti-HMGCR antibodies were strongly positive with an unremarkable paraneoplastic workup, cancer screening, and other autoimmune antibody testing. Cancer screening completed during or prior to hospitalization included prostate-specific antigen (PSA) serum testing and colonoscopy, as well as screening for the presence of common paraneoplastic antibodies that could result in other types of immune-mediated necrotizing myopathies (IMNM) (Table 1).

**Table 1:** Selected Laboratory Findings During Hospitalization *Paraneoplastic antibody panel tested for antibodies against: amphiphysin, AGNA-1, ANNA-1, ANNA-2, ANNA-3, CRMP-5, Neuronal (V-G) K+ Channel, P/Q-Type Calcium Channel, PCA-1, PCA-2, PCA-tr **Anti-ENA panel tested for antibodies against: Jo1/histidyl transfer RNA synthetase, associated with polymyositis and dermatomyositis, Ro/SSA and La/SSB, associated with Sjogren's syndrome, RNP (ribonucleoprotein), associated with mixed connective tissue disease, systemic lupus erythematous (SLE), and systemic sclerosis, Scl70/topoisomerase, associated with systemic sclerosis, and smith antibodies, associated with SLE. ALT, alanine aminotransferase; AST, aspartate aminotransferase; CK, creatine kinase; HMGCR, HMG-CoA reductase; PSA, prostate-specific antigen; TSH, thyroid-stimulating hormone

Laboratory Test	Result	Reference Range	Interpretation
CK	6,269 U/L	26-308 U/L	Elevated
AST	207 U/L	10-40 U/L	Elevated
ALT	219 U/L	7-56 U/L	Elevated
Anti-nuclear antibody (ANA)	Negative	Negative	Normal
Paraneoplastic antibody panel*	Negative	Negative	Normal
Anti-HMGCR antibody	472.2 CU	<20 CU	Elevated
Anti-extractable nuclear antigen (ENA) antibody panel**	Negative	Negative	Normal
TSH	6.21 μIU/mL	0.4-4.5 μIU/mL	Elevated
Free T4	1.3 ng/dL	0.9-1.7 ng/dL	Normal
Basic metabolic panel and complete blood count	Within normal limits	-	Normal
PSA	<0.1 ng/mL	0.0-4.0 ng/mL	Normal
C-reactive protein (CRP)	86.6 mg/L	0.0-8.0 mg/L	Elevated
Erythrocyte sedimentation rate (ENA)	16 mm/hour	0-15 mm/hour	Elevated

**Figure 1:**
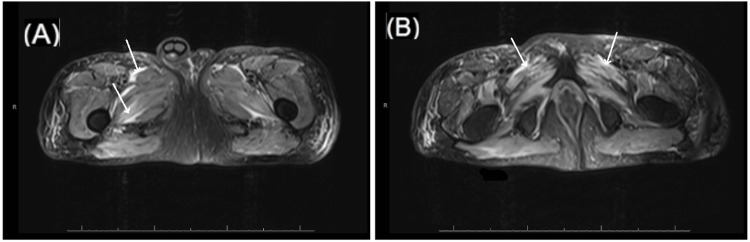
MRI Demonstrating Features of Myositis T2-weighted MRI images with contrast obtained from the patient: (A) arrows highlight hyperintensities of the adductor musculature without abnormal enhancement; (B) arrows highlight hyperintensities of the gluteus maximus musculature without abnormal enhancement. These areas of hyperintensity are consistent with patchy edema, which can be seen in myositis.

MRI of the thighs obtained prior to serology results demonstrated diffuse, symmetric T2 hyperintensity involving multiple muscle compartments, consistent with inflammatory myopathy. Images obtained during the patient’s hospitalization are included below (Figure 1). Muscle biopsy was deferred due to diagnostic serologies with imaging and laboratory findings indicative of a necrotizing myositis, consistent with SINAM.

Atorvastatin was discontinued, and high-dose prednisone (1 mg/kg/day) and methotrexate (15 mg/week) were initiated for inflammatory myopathy suppression, particularly important given the progression to symptomatic dysphagia. IVIG was deferred, given the patient's history of DVT and PE, with concern that the patient would be at high risk for thromboembolic complications of IVIG. CK declined modestly to 1,661 U/L by the time of discharge, and to 531 U/L after one month of treatment with steroids and methotrexate. While upper extremity strength minimally improved by that time, the patient remained non-ambulatory with persistent functional impairment. Transaminases also remained elevated with a smoldering CK. Due to incomplete laboratory remission with ongoing evidence of muscular necrosis and minimal functional improvement on therapy, IVIG (2 g/kg/month) was added and administered over five days to mitigate thrombotic risk. One month after IVIG, the patient achieved laboratory remission with normalized CK, AST, and ALT and improved upper extremity strength. Three months after starting IVIG, the patient began ambulating again. Eighteen months after medical treatment initiation and physical and occupational therapy, the patient had regained ambulatory function, but remains far from his baseline, reliant on a walker with persistent hip flexor weakness with 4/5 strength bilaterally and easy fatiguability. The patient is now on methotrexate 20 mg weekly, has been tapered off of prednisone, and after 12 months of IVIG 2 g/kg/month, has begun a slow IVIG taper. 

## Discussion

SINAM is a rare immune-mediated myopathy triggered after statin exposure and is distinct from self-limited statin-induced myalgia, toxicity, or rhabdomyolysis. These other forms of myositis present with mild to severe CK and transaminase elevations, often with associated myalgias. They are non-immune-mediated and resolve with withdrawal of the stain and supportive care. SINAM may initially present similarly with weakness and dysphagia with or without myalgias, but in severe cases may progress to respiratory distress [5,7]. Hypothyroid myopathy may also present similarly with proximal muscle weakness and dysphagia and should be ruled out, though it typically presents with a grossly elevated TSH, unlike our patient [8,9]. Other differentials to consider include other rheumatologic myopathies, including polymyositis, dermatomyositis, anti-SRP (signal recognition particle) IMNM, and seronegative IMNM [4,6,10], which should be ruled out with appropriate serologic testing. 

Independent risk factors for SINAM include specific HLA class II alleles (DRB111:01, DRB107:01), hypothyroidism, and malignancy [9,11,12]. If there is concern for SINAM, appropriate screening should be performed to rule out an inciting or paraneoplastic phenomenon triggering the inflammatory process as opposed to a primary drug-exposure trigger.

The pathophysiology of SINAM involves autoantibody formation against HMGCR, with muscle fiber necrosis out of proportion to inflammation, which can persist and progress after statin discontinuation [7]. Anti-HMGCR antibodies are diagnostic in the context of elevated serum CK levels and proximal muscle weakness, where antibody level correlates with severity of CK elevation and weakness [13]. Anti-HMGCR antibodies may be pathogenic in SINAM, with in vitro studies showing anti-HMGCR induced muscle fiber atrophy, inflammatory cytokine release, and impaired muscle regeneration, though more robust animal model testing is needed, as autoantibodies do not always return to normal in patients who have achieved remission [4,13]. 

While high-dose atorvastatin, 40-80 mg, is classically implicated, SINAM has been reported with low doses of other statin medications, and in patients with remote or without statin medication exposure [10,13-17]. In those who develop anti-HMGCR myositis who have not been exposed to statin medications, there is a suggested link to diets containing naturally occurring statins [4,13]. This case is the first reported case of SINAM in a patient on low-dose atorvastatin (10 mg) and underscores that a lower dosage and longer duration of exposure are not protective. In case reports, symptoms have reportedly begun between six months and nine years after statin initiation, with atorvastatin appearing to be the medication most strongly associated with SINAM [11-13,16].

Muscle biopsy, though not essential for diagnosis in seropositive cases, reveals necrotic fibers with minimal inflammatory infiltrate and macrophage-predominant infiltration [13]. MRI confirms edema and inflammation across muscle compartments and fascia (as in Figure 1), and electromyography (EMG) can show impulse fibrillations and positive sharp waves consistent with myopathic lesions [2,4,18].

Achieving remission requires immunosuppression, but treatment guidelines are limited. High-dose or pulse-dose corticosteroids are first line, with steroid-sparing agents, such as methotrexate (20-25 mg/week) and azathioprine (3 mg/kg/actual body weight), recommended as second agents with additional reported success with mycophenolate mofetil and rituximab, though optimal agents, durations, and tapers remain unclear [4,13,15]. IVIG has been shown to be effective in refractory and severe disease, alone and in combination with other immunosuppressive agents [10,15,19]. Studies suggest IVIG may be beneficial as first-line therapy for SINAM with increased frequency of partial or complete remission, though this mechanism of action is poorly understood [13]. Even with aggressive therapy, international data suggest only 42% of patients regain baseline functional status, with a 10% rate of relapse, and nearly 4% risk of mortality [3]. If IVIG is not used as initial treatment, the European Neuro Muscular Center (ENMC) recommends initiation of IVIG within six months of symptomatic treatment failure, with the suggestion to trial rituximab if there is still minimal symptomatic improvement [4]. Given our patient's failure to achieve laboratory remission or symptomatic improvement after one month of steroids and methotrexate, treatment was escalated to include IVIG through shared decision making. This patient’s clinical improvement following IVIG supports its early consideration in seropositive SINAM treatment and careful consideration of the risk of functional impairment versus thromboembolism. 

The role of the primary care provider and hospitalist is critical in early recognition of red flags like persistent serum CK elevation with proximal muscle weakness despite statin discontinuation. Referral for serologic evaluation, cancer screening, and rapid initiation of therapy is necessary to prevent progression of the disease and associated morbidity or mortality. Statins should not be reinitiated in cases of SINAM due to the immune-mediated pathophysiology of this condition and risk for relapse even with immunosuppressive therapy [4,10]. Alternative lipid-lowering strategies (e.g., PCSK9 inhibitors) may be used.

## Conclusions

SINAM is a disabling condition that requires high clinical suspicion, particularly with seemingly innocuous low-dose, chronic statin therapy. SINAM typically presents with progressive limb-girdle weakness and elevated serum CK levels after statin exposure. Anti-HMGCR antibodies are key for diagnosis, which is also supported by characteristic findings on muscle biopsy, MRI, and EMG. Early immunosuppressive therapy, including corticosteroids, steroid-sparing oral agents, and IVIG, can be effective treatments, with IVIG likely contributing most significantly to functional recovery. This patient required treatment with IVIG before experiencing any significant functional recovery, highlighting the importance of escalation to IVIG in refractory cases of SINAM and suggesting that early IVIG treatment may be useful in cases with significant functional impairment. Given the risk for significant debility, clinicians must maintain a high index of suspicion for SINAM despite duration or dose of statin therapy in order to make an appropriate diagnosis and ensure timely treatment. Primary care clinicians play a particularly important role in diagnosis before significant debility develops, given the chronic and insidious onset of SINAM.
